# Dental implant rehabilitation in a patient with primary hyperoxaluria type 1: a 14-month case report

**DOI:** 10.1186/s12903-026-08939-7

**Published:** 2026-06-18

**Authors:** Abdalla R. Sulaiman, Ghassan E. Bassit

**Affiliations:** 1 Department of Oral Surgery, Faculty of Dentistry, International University for Science and Technology, Damascus, Syria; 2 Resident Doctor, Ministry of Health, Damascus, Syria

**Keywords:** Primary hyperoxaluria type 1, Oxalosis, Dental implants, Osseointegration, Periodontal manifestations, Oral rehabilitation, Implant stability quotient (ISQ)

## Abstract

**Background:**

Primary Hyperoxaluria Type 1 (PH1) is a rare metabolic disorder characterized by systemic oxalate deposition, which may influence bone metabolism through mechanisms such as oxalate accumulation in alveolar bone, renal osteodystrophy, and the long‑term effects of immunosuppressive therapy following transplantation. In the present case, conventional prosthetic options were unsuitable due to external root resorption and the poor prognosis of the remaining dentition.

**Case presentation:**

This report describes implant‑supported oral rehabilitation in a medically complex patient with PH1 and a history of combined liver–kidney transplantation and multiple systemic comorbidities. Dental implant therapy was selected following comprehensive clinical and radiographic assessment. At six months, resonance frequency analysis demonstrated secondary stability with ISQ values of 75 (right) and 74 (left). Clinical and radiographic evaluation at 14 months showed stable peri‑implant conditions without detectable complications.

**Conclusion:**

This case suggests that implant therapy may be feasible in selected patients with PH1 when thorough risk assessment, individualized planning, and close postoperative monitoring are ensured. However, the findings are limited by the single‑case design and relatively short follow‑up period, and should be interpreted as hypothesis‑generating rather than conclusive evidence regarding long‑term implant outcomes in this population.

## Introduction

Primary hyperoxaluria type 1 (PH1) is a rare autosomal recessive disorder caused by deficiency of the hepatic enzyme alanine‑glyoxylate aminotransferase, leading to hepatic overproduction of oxalate and systemic deposition of calcium oxalate crystals (oxalosis) [1, 2]. Clinically, PH1 is characterized by recurrent nephrolithiasis, nephrocalcinosis, and progressive renal impairment, often culminating in end‑stage renal disease (ESRD) [3, 4]. The systemic burden of oxalosis extends beyond the kidneys, affecting bones, eyes, skin, and cardiovascular tissues, thereby contributing to significant morbidity and mortality [5–7].

Management of PH1 remains challenging. Conservative measures such as high fluid intake and pyridoxine supplementation may slow disease progression, but many patients ultimately require renal replacement therapy or combined liver–kidney transplantation to correct the underlying metabolic defect [8, 9]. International registries have highlighted substantial variability in disease onset and progression, ranging from early childhood presentations to adult diagnoses at advanced stages [4].

Oral and maxillofacial manifestations of PH1 have been increasingly recognized. Reported findings include periodontal breakdown, jawbone fragility, external root resorption, mucosal ulcerations, and gingival discoloration associated with oxalate deposition [10–12]. Histopathological studies have confirmed calcium oxalate crystals within the periodontium, alveolar bone marrow, and gingival tissues, provoking chronic inflammation and contributing to tissue destruction [13, 14]. Severe periodontitis, osteomalacia‑related bone fragility, and soft tissue alterations have also been documented, complicating oral rehabilitation in affected patients [15–17].

Dental implant therapy relies on adequate bone quality, controlled inflammation, and predictable healing—factors that may be impaired in PH1 due to systemic oxalosis, renal dysfunction, and the potential effects of long‑term immunosuppressive therapy in post‑transplant patients [18]. Careful assessment of implant stability, including implant stability quotient (ISQ) measurements, has been recommended in medically complex individuals to support clinical decision‑making [19]. Systemic diseases and long‑term medications have also been shown to influence osseointegration and peri‑implant health, underscoring the importance of individualized assessment in medically compromised patients [20].

Despite the growing recognition of oral complications in PH1, evidence regarding implant therapy in this population remains extremely limited. Most published reports focus on periodontal or orthodontic management rather than implant‑based rehabilitation [16, 17]. Documenting implant outcomes in PH1 patients is therefore essential to expand current knowledge and guide future treatment strategies.

The aim of this case report is to describe the clinical, radiographic, and functional outcomes of implant‑supported rehabilitation in a medically complex patient with PH1, highlighting diagnostic considerations, surgical planning, and short‑term follow‑up in the context of systemic oxalosis.

## Case presentation

### Patient information

A 23-year-old male diagnosed with Primary Hyperoxaluria Type 1 (PH1) since childhood and previously monitored at Birmingham Children’s Hospital and University Hospital Birmingham presented to a private dental clinic with the chief complaint of missing teeth and impaired mastication. His medical history was significant for two kidney transplants, a liver transplant, pulmonary hypertension, unexplained pancytopenia, a single-gland parathyroidectomy, and a previous hip fracture attributed to systemic oxalosis. At the time of dental evaluation, his systemic condition was stable under multidisciplinary follow-up. Current medications included tacrolimus, prednisolone, aspirin, vitamin D, omeprazole, and a vitamin B complex (Table 1).

**Table 1:** Summary of systemic conditions, medications, laboratory findings, and medical risk assessment

Category	Findings	Clinical Relevance
Systemic Conditions	Primary Hyperoxaluria Type 1; history of combined liver–kidney transplantation; pulmonary hypertension; chronic immunosuppression; pancytopenia.	Increased risk of infection, altered bone metabolism, and delayed healing.
Immunosuppressive Medications	Tacrolimus; Mycophenolate mofetil; Prednisone.	Potential effects on osteoblastic activity and osseointegration.
Other Medications	Antihypertensives; Vitamin D supplementation.	May influence bone turnover and systemic stability.
Laboratory Values	Hemoglobin: 11.2 g/dL; WBC: 3.8 × 10⁹/L; Platelets: 92 × 10⁹/L; Creatinine: 1.3 mg/dL; Liver enzymes: within acceptable post-transplant range.	Platelet count required for bleeding-risk assessment; overall labs indicate stable transplant function.
Bleeding Risk Assessment	Mild thrombocytopenia without clinical bleeding tendency; no coagulopathy.	Supports safe implant placement under controlled conditions.
Infection Risk Assessment	Chronic immunosuppression; transplant team recommended perioperative antibiotics.	Justifies perioperative antibiotic coverage.
Airway / Anesthesia Considerations	No contraindications to local anesthesia.	Safe for outpatient implant surgery.
Overall Medical Risk Classification	ASA III	Requires modified treatment planning and close postoperative monitoring.

### Medical risk assessment

Given the patient’s complex systemic background, a structured medical risk assessment was performed in accordance with CARE guidelines. A formal consultation with the transplant team confirmed stable graft function and no contraindications to elective dental surgery. Recent laboratory investigations demonstrated acceptable renal function for a post-transplant patient, normal serum calcium and phosphate levels, and no evidence of acute metabolic imbalance. The patient’s immunosuppressive regimen was reviewed to assess infection risk and potential effects on bone metabolism. Prophylactic antibiotic coverage was planned due to chronic immunosuppression. No drug interactions with local anesthetics or perioperative medications were identified.

### Clinical and radiographic findings

**Fig. 1:**
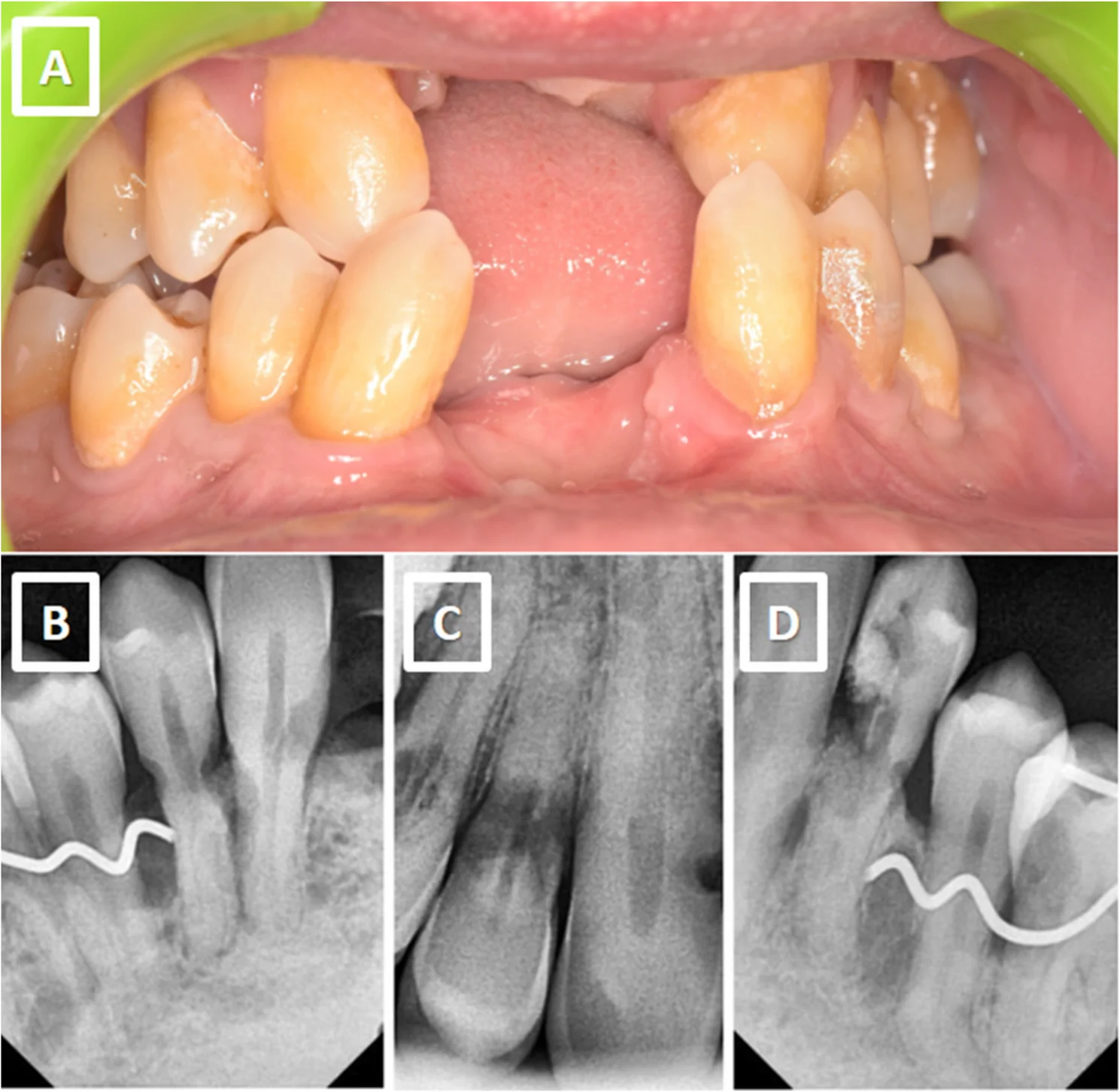
**A** Intraoral photograph at initial examination showing missing teeth and malocclusion. **B**–**D** Periapical radiographs demonstrating external root resorption with variable morphologies

Comprehensive extraoral and intraoral examinations were performed. The patient exhibited loss of the maxillary and mandibular incisors and bilateral maxillary first molars. Baseline periodontal evaluation revealed a plaque index of 20% (Silness–Löe Plaque Index) and a gingival index of 1.2 (Löe–Silness Gingival Index), with probing depths of 2–3 mm and no active periodontal pockets. The oral mucosa, tongue, and gingiva appeared clinically normal without ulcerations or discoloration (Fig. 1).

Occlusal analysis demonstrated an anterior open bite and reduced posterior support. Study models confirmed compromised occlusal stability and limited restorative options.

**Fig. 2:**
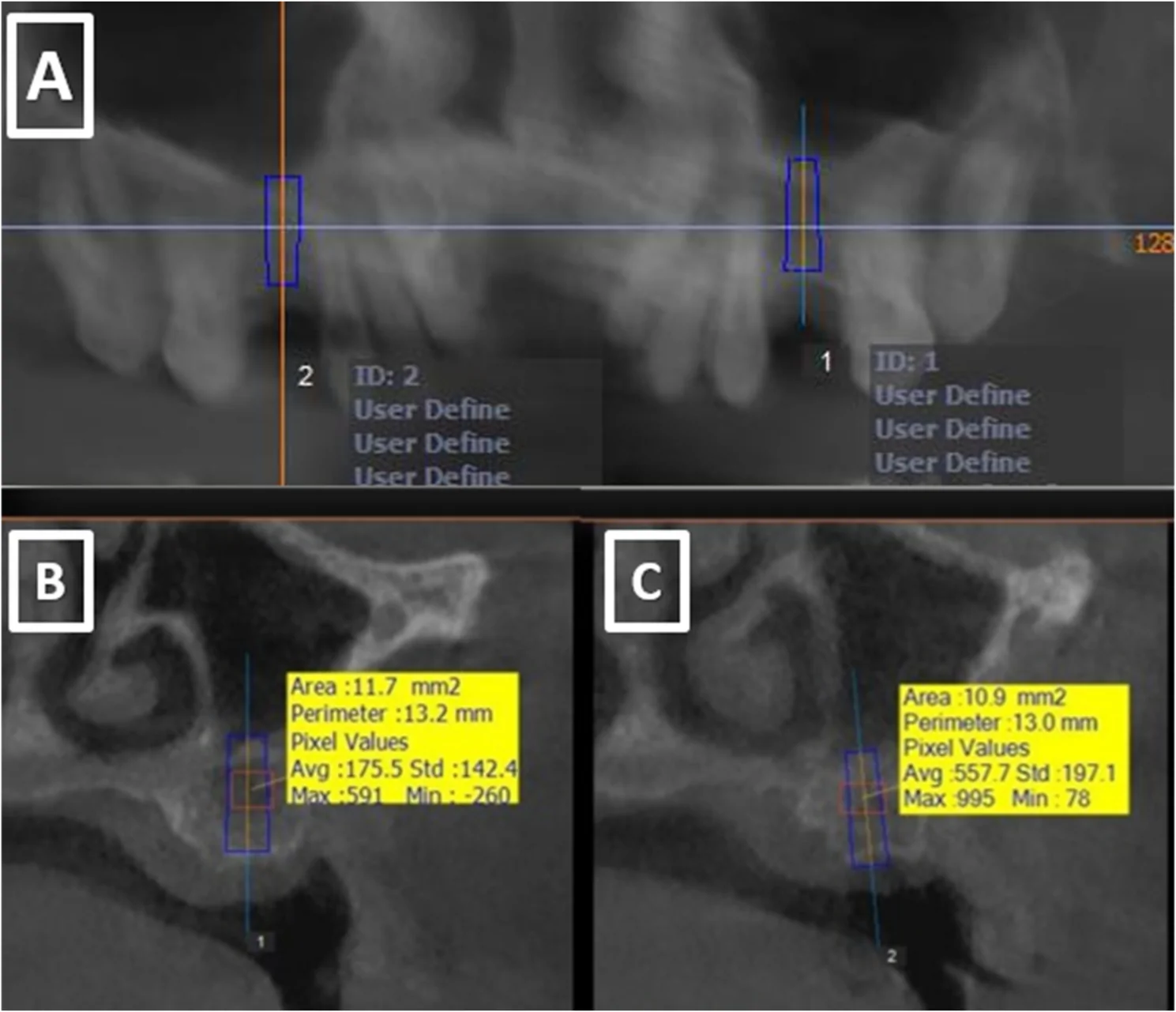
**A** Preoperative panoramic CBCT reconstruction illustrating edentulous areas planned for implant placement. **B**, **C** Cross-sectional CBCT views showing bone dimensions and density at the two implant sites

Cone beam computed tomography (CBCT) revealed reduced bone volume in the anterior maxilla and mandible, with adequate bone height in the maxillary first molar regions. Bone density measurements were 175.5 voxel values on the left posterior maxilla and 557.7 voxel values on the right side (Fig. 2). Given the known variability and limited reliability of CBCT-derived grayscale values, these measurements were interpreted cautiously and supplemented with clinical assessment. Bone quality was classified as Misch D4 on the left side and D3 on the right side. No sinus pathology or anatomical limitations were identified.

### Treatment planning

A staged approach was adopted. Two implants were planned for the maxillary first molar regions to evaluate healing and stability before proceeding with anterior and additional implant sites. The treatment plan prioritized minimizing surgical trauma, ensuring controlled loading, and maintaining strict postoperative monitoring due to the patient’s systemic condition.

### Surgical procedure

The surgical field was disinfected with povidone-iodine and rinsed with sterile saline. Local anesthesia was administered using posterior superior alveolar, middle superior alveolar, and greater palatine nerve blocks with 2% lidocaine containing 1:80,000 adrenaline. Antibiotic prophylaxis was administered based on explicit recommendation from the transplant team, given the patient’s chronic immunosuppression and overall medical complexity. This approach reflects individualized medical guidance rather than routine prophylaxis for immunosuppression alone, in accordance with current AAOMS and AHA recommendations.

**Fig. 3:**
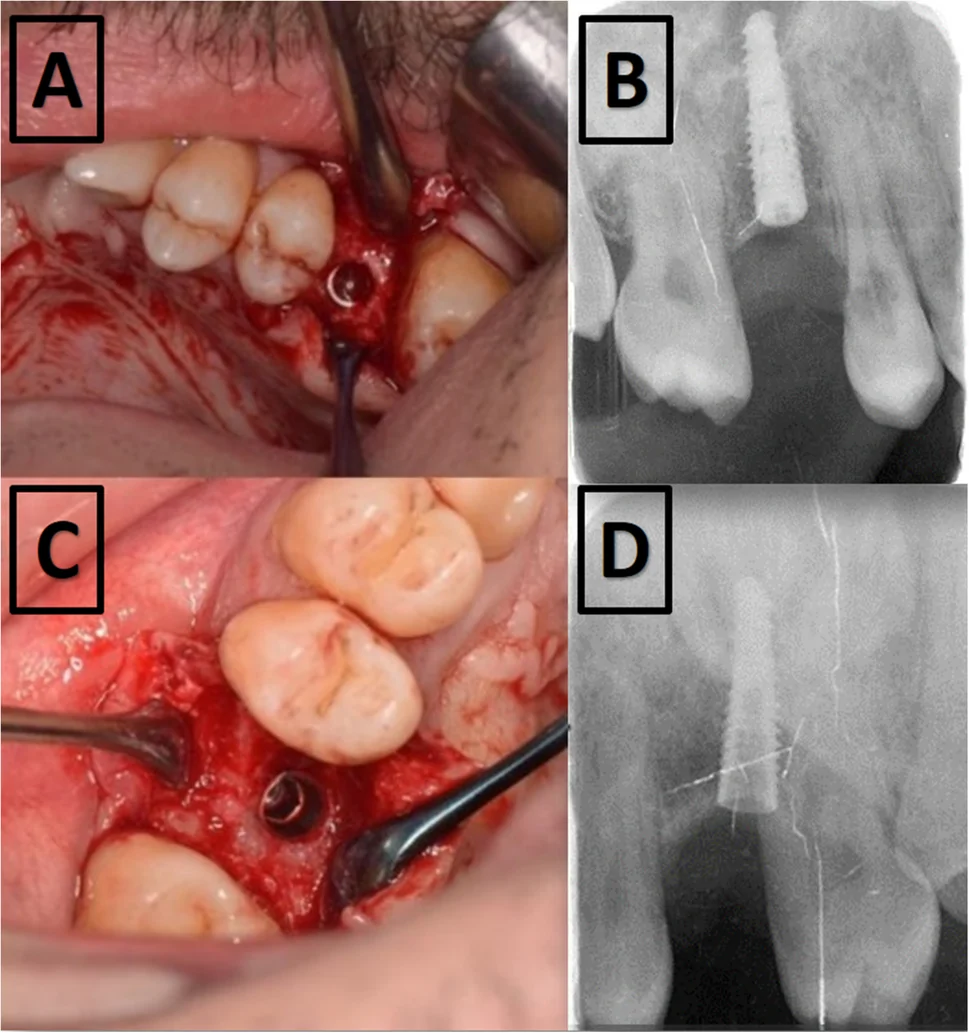
**A**, **C** Clinical views of left and right implants seated within alveolar bone. **B**, **D** Corresponding periapical radiographs immediately after placement

A full-thickness mucoperiosteal flap was elevated. The drilling protocol followed the manufacturer’s recommendations with slight undersizing to enhance primary stability in D3–D4 bone. Sequential drilling was performed under copious sterile saline irrigation. Two internal hex implants (NEODENT Implant System, Curitiba, Brazil), each measuring 4 × 10 mm, were placed in the maxillary first molar regions (Fig. 3). Insertion torque values ranged between 30 and 35 Ncm.

Resonance frequency analysis (RFA) was not recorded at the time of surgery due to the unavailability of the device during the surgical appointment; however, primary stability was clinically satisfactory based on insertion torque and tactile assessment.

Healing was uneventful, with no postoperative pain or complications. Sutures were removed after 10 days, and follow-up visits were conducted at 1 and 2 months.

### Prosthetic phase and outcomes

**Fig. 4:**
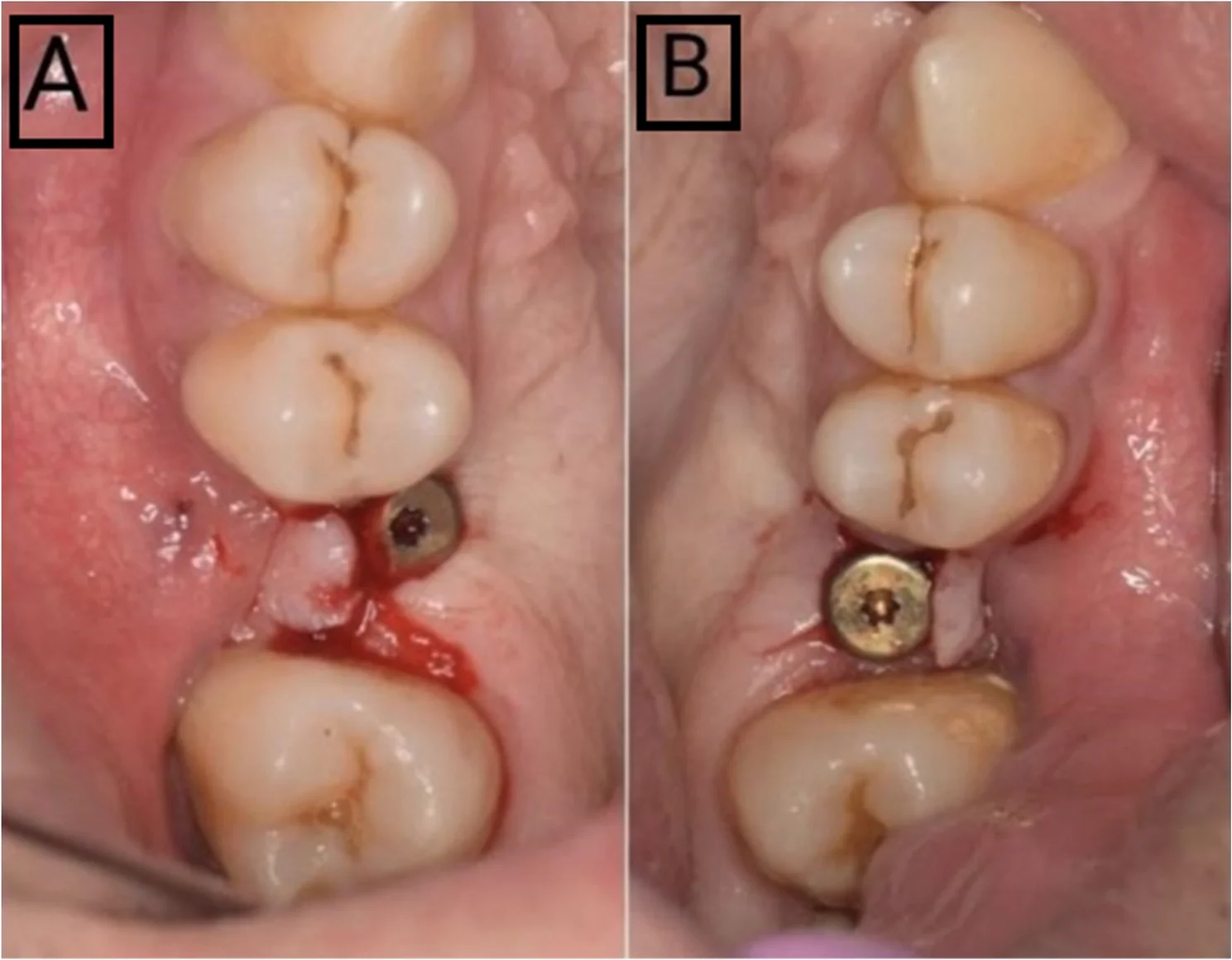
Clinical views showing uncovering of implants and placement of healing abutments on the (**A**) right and (**B**) left sides

**Fig. 5:**
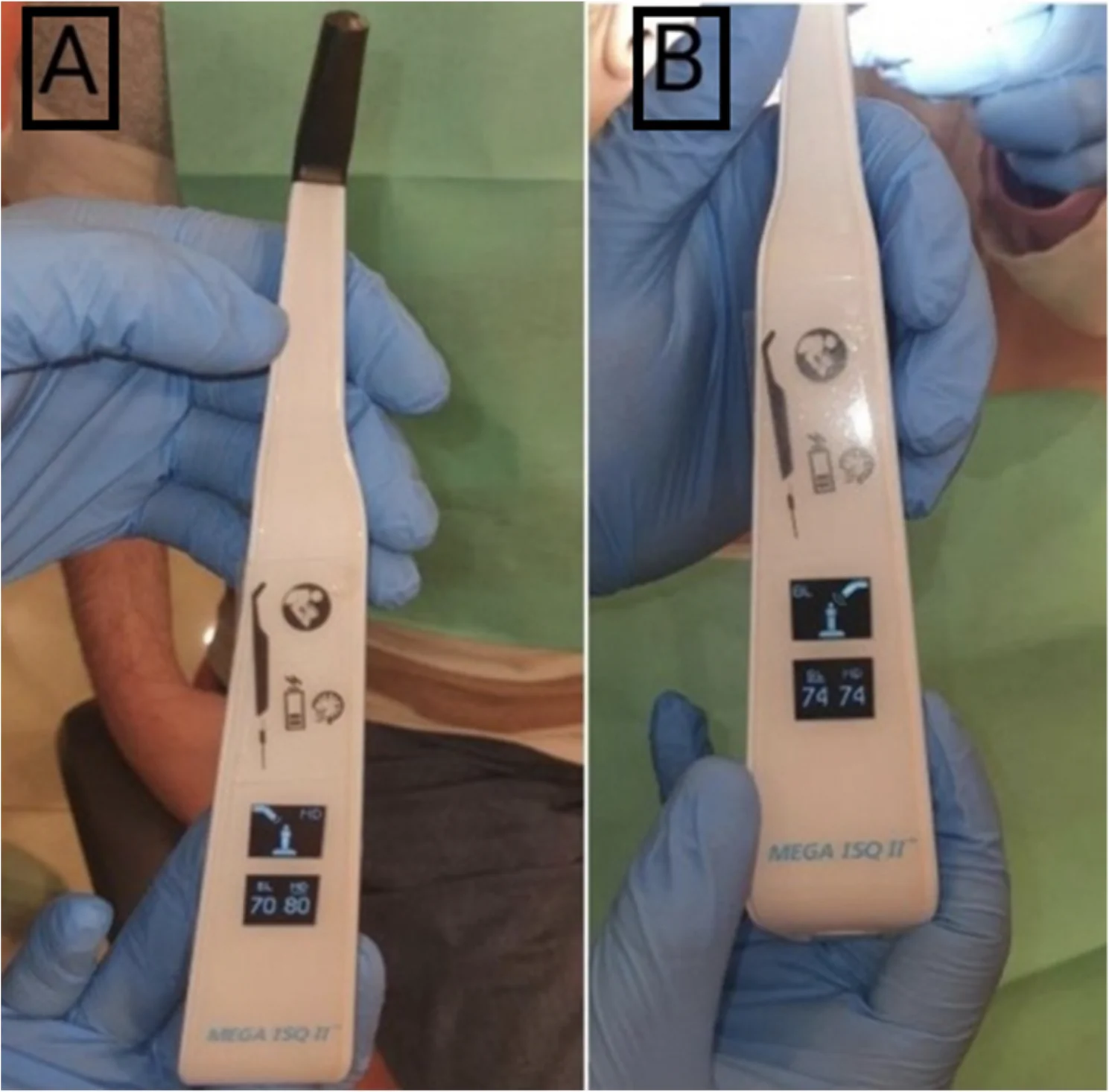
Implant Stability Quotient (ISQ) values measured at the (**A**) right and (**B**) left implant sites

Six months post-surgery, the implants were uncovered and healing abutments placed (Fig. 4). Fifteen days later, prosthetic rehabilitation commenced. Implant stability was assessed using resonance frequency analysis (MEGA ISQ II, MegaGen, Seoul, Korea). According to manufacturer guidelines, ISQ values above 70 indicate high stability, while values between 60 and 69 indicate medium stability [21, 22]. The right implant recorded an ISQ of 75 and the left implant 74, indicating favorable secondary stability (Fig. 5).

**Fig. 6:**
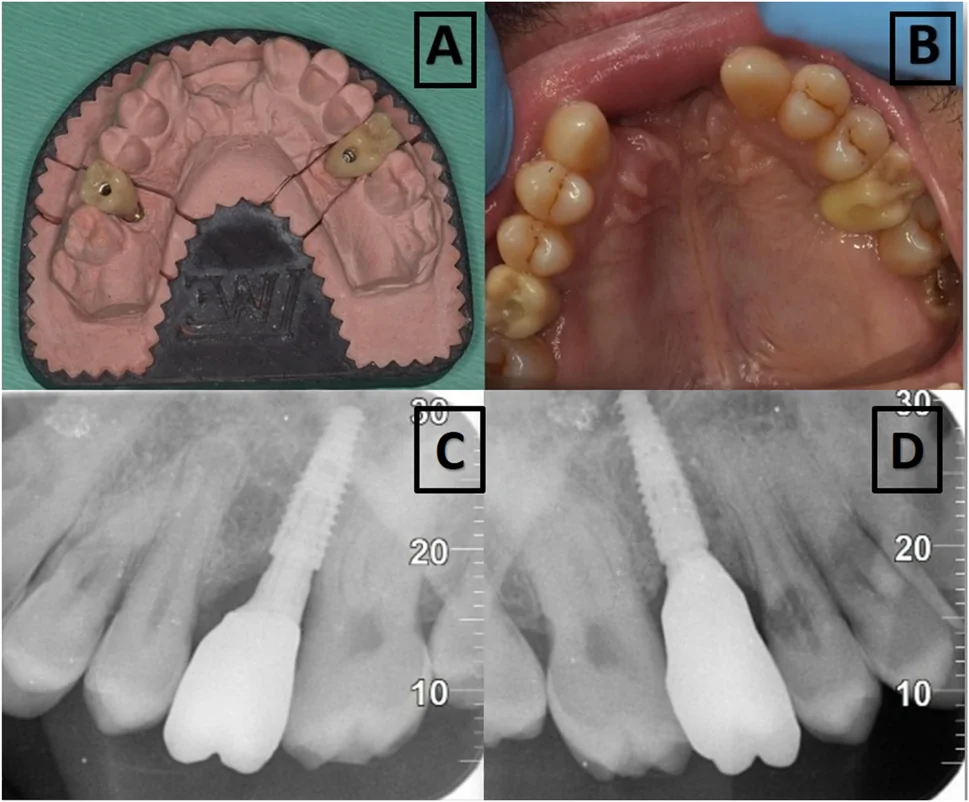
**A** Final crowns on laboratory model. **B** Clinical view after screw-retained crown placement. **C**, **D** Periapical radiographs showing precise fit of the final crowns with no detectable peri-implant bone loss

Final zirconia restorations were screw-retained to minimize risks associated with cement remnants. Radiographs confirmed accurate crown fit and the absence of peri-implant radiolucency (Fig. 6).

### Follow-up

The patient remained stable throughout the healing and prosthetic phases. At the 14-month follow-up, both implants demonstrated functional success, with no mobility, no suppuration, no peri-implant radiolucency, and no soft-tissue inflammation detected on routine clinical examination.

Quantitative marginal bone level measurements were performed using linear assessment from the implant–abutment interface to the first bone-to-implant contact on both mesial and distal aspects. Baseline measurements obtained from the immediate postoperative periapical radiographs (Fig. 3) were compared with the 14-month follow-up radiographs (Fig. 6). The right implant demonstrated a mesial change of approximately 0.2 mm and a distal change of 0.1 mm, while the left implant showed a mesial change of 0.1 mm and a distal change of 0.2 mm. These minimal variations fall within the expected range of early crestal bone remodeling and do not indicate pathological bone loss.

Qualitative radiographic evaluation further demonstrated stable crestal bone levels without peri-implant radiolucency throughout the 14-month follow-up period. Long-term monitoring is ongoing, and extended outcomes will be reported in future assessments 

## Discussion

Rehabilitation of patients with Primary Hyperoxaluria Type 1 (PH1) requires careful consideration due to the systemic effects of oxalate deposition on bone metabolism and soft tissues. Although oral manifestations such as periodontal breakdown, external root resorption, and compromised bone quality have been described in PH1 [13–17], their implications for implant therapy remain insufficiently explored. In the present case, implant-supported rehabilitation was selected after alternative options were deemed unsuitable due to the poor prognosis of the remaining dentition.

A major factor influencing treatment planning was the patient’s complex medical history, including prior liver and kidney transplantation, chronic immunosuppressive therapy, pulmonary hypertension, and pancytopenia. Immunosuppressive medications—particularly calcineurin inhibitors and long-term corticosteroids—have been associated with altered bone turnover, reduced osteoblastic activity, and delayed wound healing, which may influence osseointegration [23]. Despite these concerns, implant therapy in medically compromised or transplant patients has been shown to be feasible when systemic conditions are stable and appropriate maintenance protocols are implemented. Large cohort studies indicate that systemic factors such as immunosuppression, renal disease, and metabolic disorders may increase the risk of complications but do not necessarily contraindicate implant placement [24, 25]. Similarly, metabolic bone diseases—including renal osteodystrophy and osteoporosis—have been associated with reduced bone density and altered healing capacity, yet successful implant outcomes have been reported when careful case selection and controlled loading protocols are applied [26].

In the present case, the implants demonstrated adequate secondary stability, with ISQ values of 75 and 74 at six months. While higher ISQ values are generally associated with favorable secondary stability, such findings should be interpreted cautiously in a single-patient case report and cannot be extrapolated to predict long-term outcomes [27]. Radiographic evaluation revealed stable peri-implant bone levels without radiolucency, and clinical examination confirmed healthy peri-implant soft tissues throughout the 14-month follow-up period. However, the absence of standardized peri-implant parameters—such as probing depths, bleeding on probing, and quantitative marginal bone level measurements—limits the ability to fully characterize peri-implant tissue health.

It is also important to acknowledge that systemic risks associated with PH1—such as oxalate deposition in alveolar bone, renal osteodystrophy, and the long-term effects of calcineurin inhibitors and corticosteroids—may manifest over extended periods, particularly beyond 5–10 years. Therefore, the absence of complications at 14 months should be interpreted with caution, and long-term follow-up is essential to determine implant behavior in this population.

Recent advances in artificial intelligence (AI) have introduced new tools for evaluating biological and microbiological parameters in medically complex patients. AI-assisted analysis of peri-implant microbiota and tissue health has shown potential in improving diagnostic accuracy and treatment planning. Although such technologies were not used in the present case, they may offer valuable support in future assessments of patients with systemic metabolic disorders, including PH1.

Overall, this case contributes to the limited evidence regarding implant therapy in PH1 and highlights the importance of individualized planning, multidisciplinary coordination, and cautious interpretation of short-term outcomes. Further studies with standardized diagnostic protocols and long-term follow-up are needed to clarify the impact of PH1 and associated systemic conditions on implant survival and peri-implant tissue stability.

## Conclusion

This case illustrates that implant-supported rehabilitation may be feasible in selected patients with Primary Hyperoxaluria Type 1 when comprehensive medical evaluation, multidisciplinary coordination, and carefully controlled clinical conditions are ensured. However, the findings should not be interpreted as a general endorsement of implant therapy in PH1 patients, as the evidence remains limited to a single-case report with a relatively short follow-up period. The outcomes presented here should be viewed as hypothesis-generating rather than conclusive, and long-term studies are required to better understand implant behavior and peri-implant tissue stability in this medically complex population.

## Data Availability

All data generated or analyzed during this case report are included in this published article.
